# Indoor Firing Ranges and Elevated Blood Lead Levels — United States, 2002–2013

**Published:** 2014-04-25

**Authors:** Catherine Beaucham, Elena Page, Walter A. Alarcon, Geoffrey M. Calvert, Mark Methner, Todd M. Schoonover

**Affiliations:** 1 Div of Surveillance, Hazard Evaluations, and Field Studies, National Institute for Occupational Safety and Health, CDC; 2 Washington State ABLES Program, Washington State Department of Labor and Industries

Indoor firing ranges are a source of lead exposure and elevated blood lead levels (BLLs) among employees, their families, and customers, despite public health outreach efforts and comprehensive guidelines for controlling occupational lead exposure (1). There are approximately 16,000–18,000 indoor firing ranges in the United States, with tens of thousands of employees. Approximately 1 million law enforcement officers train on indoor ranges (1). To estimate how many adults had elevated BLLs (≥10 *μ*g/dL) as a result of exposure to lead from shooting firearms, data on elevated BLLs from the Adult Blood Lead Epidemiology and Surveillance (ABLES) program managed by CDC’s National Institute for Occupational Safety and Health (NIOSH) were examined by source of lead exposure. During 2002–2012, a total of 2,056 persons employed in the categories “police protection” and “other amusement and recreation industries (including firing ranges)” had elevated BLLs reported to ABLES; an additional 2,673 persons had non–work-related BLLs likely attributable to target shooting. To identify deficiencies at two indoor firing ranges linked to elevated BLLs, the Washington State Division of Occupational Safety and Health (WaDOSH) and NIOSH conducted investigations in 2012 and 2013, respectively. The WaDOSH investigation found a failure to conduct personal exposure and biologic monitoring for lead and also found dry sweeping of lead-containing dust. The NIOSH investigation found serious deficiencies in ventilation, housekeeping, and medical surveillance. Public health officials and clinicians should ask about occupations and hobbies that might involve lead when evaluating findings of elevated BLLs. Interventions for reducing lead exposure in firing ranges include using lead-free bullets, improving ventilation, and using wet mopping or high-efficiency particulate air (HEPA) vacuuming to clean (1).

## ABLES Data, 2002–2012

In 2012, 41 states participated in ABLES, receiving notification from laboratories and physicians of elevated BLLs in persons aged ≥16 years through reporting mandated by state laws (2). Only the highest BLL was included if more than one was collected within a single year from an individual. Workers in the categories “police protection” or “other amusement and recreation industries” (OARI), which includes firing ranges, were considered to have occupational lead exposures.

During 2002–2012, a total of 2,056 persons in the two industry categories had BLLs ≥10 *μ*g/dL; 785 had BLLs ≥25 *μ*g/dL, and 1,271 had BLLs of 10–24 *μ*g/dL. Of the 2,056, a total of 631 (31%) were employed in police protection, and 1,425 (69%) were employed in OARI (Table 1). During 2002–2012, non–work-related target shooting was the likely exposure for an additional 2,673 persons with elevated BLLs (1,290 with BLLs ≥25 *μ*g/dL and 1,388 with BLLs of 10–24 *μ*g/dL).

## WaDOSH Investigation, 2012

In 2010, the Washington state ABLES program requested an inspection by WaDOSH of an indoor firing range after seven employees were found to have elevated BLLs. WaDOSH issued citations for violations of seven sections of their workplace lead standard, which is identical to the federal Occupational Safety and Health Administration (OSHA) standard.

In October 2012, the state ABLES program received reports of BLLs of 40 *μ*g/dL and 48 *μ*g/dL in two employees of the same range. Interviews revealed ongoing renovation at the range beginning in September 2012, including replacing the sand berm bullet trap with a steel bullet trap, replacing the ventilation system, and adding a second floor. Review of records revealed that from 2010 until the onset of renovation, 19 range employees had BLLs of 12–50 *μ*g/dL. Following initial ABLES interviews, a compliance inspection from WaDOSH was conducted.

In the 2012 inspection, WaDOSH noted the ventilation system was inoperable and temporarily replaced by two roof fans that exhausted unfiltered air outside. Multiple citations were issued for violations of the workplace lead standard, including failure to conduct personal exposure and biologicmonitoring for lead, dry sweeping of lead-containing dust, and lack of respirator medical clearance and fit testing.

During renovation of the firing range, 117 construction workers and 42 range employees were present. A total of 98 of these persons received BLL testing, and 46 (47%) had elevated BLLs, including 26 construction workers (BLLs of 10–153 *μ*g/dL) and 20 range employees (BLLs of 14–58 *μ*g/dL). The BLL of 153 *μ*g/dL was recorded approximately 10 weeks after the construction worker began dismantling the frame of the sand berm and installing the steel bullet trap. Interviews with nine construction workers and six range employees with BLLs ≥40 *μ*g/dL documented inadequate knowledge regarding the hazards of workplace and “take-home” lead exposures (e.g., lead transferred to family members via clothing or automobile interiors). As a result of this investigation, WaDOSH initiated standardized inspections of all firing ranges in the state, including exposure monitoring and lead safety training for firing range employees.

What is already known on this topic?

Guidelines for the management of lead-exposed adults at or above the current CDC reference blood lead level (BLL) of 10 *μ*g/dL are available. Despite public health outreach and comprehensive guidelines for controlling lead exposure in indoor firing ranges, these ranges continue to be a prominent source of lead exposure and elevated BLLs.

What is added by this report?

Data collected by the Adult Blood Lead Epidemiology and Surveillance program in 41 states during 2002–2012 identified 2,056 persons with BLLs ≥10 *μ*g/dL who were likely exposed to firearms at work and an additional 2,673 persons likely exposed by non–work-related target shooting. Two investigations highlight the nature of lead exposure in firing ranges.

What are the implications for public health practice?

Employees and customers of indoor firing ranges, and their family members, continue to be exposed to hazardous amounts of lead. Lead exposures in firing ranges can be reduced by improving ventilation systems, use of wet mopping or high-efficiency particulate air vacuuming to remove dust and debris, and use of lead-free bullets. Public health practitioners, state and government agencies, and community organizations should be encouraged to increase lead exposure prevention efforts directed at employers, employees, and the community.

The state ABLES program advised employees to have family members tested; three children and two adult family members of four construction workers had BLLs ≥5 *μ*g/dL. Positive tests for surface lead contamination in homes and vehicles of several workers required lead abatement from hard surfaces, carpeting, and upholstery. A recreational shooter at the range reported a BLL of 12.9 *μ*g/dL to public health authorities.

## NIOSH Investigation, 2013

In December 2013, at the request of employees, NIOSH investigators evaluated lead exposure at an indoor firing range and firearms retailer in California. Investigators reviewed medical and exposure records, interviewed five of the six employees, collected air and surface wipe samples for lead, and evaluated the ventilation systems for the range and showroom. Employees spent most of their work day on the sales floor or in the office, entering the range generally to assist shooters experiencing difficulty. Employees cleaned the range daily using a floor squeegee for spent bullet casings and a HEPA-filtered vacuum cleaner on carpeted areas. They replaced filters in the range exhaust ventilation system and scraped and oiled the steel bullet trap weekly.

Numerous deficiencies were found (Table 2). Six full-shift personal air samples from monitors worn by showroom employees had lead concentrations of 5.5–19 *μ*g/m^3^, within the current OSHA occupational exposure limit of 50 *μ*g/m^3^. Two task-based air samples for lead had high short-term (<1 hour) concentrations of 54 *μ*g/m^3^ (for nightly range maintenance) and 64 *μ*g/m^3^ (for weekly range cleaning). Lead was detected on all surfaces tested. Employee BLL testing had been conducted for the first time immediately before the NIOSH evaluation, and BLLs ranged from 19.9 *μ*g/dL to 40.7 *μ*g/dL. No employees had undergone other medical surveillance as required by the California Division of Occupational Safety and Health and OSHA (3). Recommendations were made to minimize employee and customer exposure to lead, and the county public health officer was notified regarding risks to customers from airborne and surface lead exposure. Employees were advised to send family members for BLL testing because of the potential for take-home lead exposures.

### Discussion

The ABLES data and the two investigations summarized in this report document serious lead exposure from indoor firing ranges (4). Employers in general industry are required by law to follow the OSHA lead standard established in 1978 (3,5). OSHA considers the permissible airborne lead exposure limit of 50 *μ*g/m^3^ and allowable BLLs to be outdated (5,6).* The National Toxicology Program recently released a monograph on the potential health effects of low-level lead exposure to adults (7) (Table 3).

In 2013, the California Department of Public Health recommended that the California Division of Occupational Safety and Health lower the permissible exposure limit for lead in air to 0.5–2.1 *μ*g/m^3^ to keep BLLs below the range of 5–10 *μ*g/dL (8). Guidelines for management of lead exposed employees (9) are endorsed by the California Department of Public Health, the Council of State and Territorial Epidemiologists, and the American College of Occupational and Environmental Medicine, and recommended by NIOSH (1). Importantly, these guidelines are not based on airborne lead levels, but on monitoring BLLs, which can reflect exposure through any route. BLLs should be kept below 10 *μ*g/dL for all adults, and below 5 *μ*g/dL for children and pregnant women (9).

The findings in this report also suggest that firing range customers and family members of firing range employees, in addition to employees themselves, can be exposed to hazardous amounts of lead. There are an estimated 19 million active target shooters in the United States (10).

The findings in this report are subject to at least five limitations. First, employers might not provide BLL testing to all lead-exposed employees as required. Second, adults with non–work-related exposures are not likely to be tested, and BLLs of recreational shooters are not consistently available. Third, certain laboratories might not report BLL test results as required. Fourth, how many of the elevated BLLs were related to firing range exposures is not known. Because the OARI industry category includes industries other than firing ranges (e.g., miniature golf courses and billiard parlors), it is possible that some OARI workers with occupational BLL elevations were not employed in firing ranges. Finally, the two investigations did not determine the full extent of take-home exposures and other sources of lead exposure among firing range workers and customers.

The number of persons with elevated BLLs from firearms use during 2011–2012 highlights the need to increase prevention activities. Airborne and surface lead levels in firing ranges can be greatly reduced by using lead-free bullets, improving ventilation systems, using wet mopping or HEPA vacuuming instead of dry sweeping, and having a written protocol for range maintenance (1). Measures also should be taken to prevent take-home exposure.†

## Figures and Tables

**TABLE 1:** Number and percentage* of adults with elevated blood lead levels (≥10 *μ*g/dL), by selected categories — Adult Blood Lead Epidemiology and Surveillance (ABLES) program, United States, 2002–2012

Category	2002		2003		2004		2005		2006		2007		2008		2009		2010		2011		2012		Total
											
	No.	(%)	No.	(%)	No.	(%)	No.	(%)	No.	(%)	No.	(%)	No.	(%)	No.	(%)	No.	(%)	No.	(%)	No.	(%)	No.
**Adults with work-related exposures from firearm use, by industry subsector**																							
Police Protection, NAICS code 92212																							
BLL ≥25 *μ*g/dL	21	(0.3)	16	(0.2)	5	(0.1)	13	(0.2)	6	(0.1)	11	(0.2)	9	(0.1)	14	(0.3)	15	(0.2)	9	(0.1)	13	(0.2)	**132**
BLL 10–24 *μ*g/dL	19	(0.3)	16	(0.2)	21	(0.3)	24	(0.3)	40	(0.5)	45	(0.6)	67	(0.7)	75	(0.8)	67	(0.5)	71	(0.5)	54	(0.4)	**499**
All Other Amusement and Recreation Industries, NAICS 71399 (including firing ranges)																							
BLL ≥25 *μ*g/dL	41	(0.6)	43	(0.6)	31	(0.5)	47	(0.8)	50	(0.7)	47	(0.7)	43	(0.6)	43	(0.8)	38	(0.6)	125	(1.8)	145	(2.5)	**653**
BLL 10–24 *μ*g/dL	15	(0.2)	18	(0.3)	24	(0.3)	51	(0.7)	43	(0.5)	58	(0.7)	71	(0.8)	64	(0.7)	91	(0.7)	125	(0.9)	212	(1.6)	**772**
Total exposed at work (including non–firearm-related exposures)																							
BLL ≥25 *μ*g/dL	6,768	—	7,194	—	6,496	—	5,545	—	6,878	—	6,625	—	6,657	—	5,351	—	6,882	—	6,890	—	5,793	—	**71,079**
BLL 10–24 *μ*g/dL	7,390	—	6,396	—	7,133	—	7,656	—	7,821	—	7,888	—	9,026	—	9,355	—	12,211	—	14,093	—	13,140	—	**101,962**
**Adults with non–work-related exposures from firearm use**																							
Target shooting																							
BLL ≥25 *μ*g/dL	98	(24.9)	100	(27.8)	95	(31.3)	98	(30.2)	131	(34.0)	121	(34.0)	123	(35.9)	103	(30.4)	138	(39.4)	136	(33.8)	147	(37.5)	**1,290**
BLL 10–24 *μ*g/dL	33	(18.4)	56	(24.8)	79	(25.8)	71	(26.3)	70	(21.0)	87	(20.8)	75	(17.3)	160	(28.7)	188	(25.5)	272	(31.3)	292	(38.2)	**1,383**
Total not exposed at work (including non–firearm-related exposures)																							
BLL ≥25 *μ*g/dL	393	—	360	—	304	—	325	—	385	—	356	—	343	—	339	—	350	—	402	—	392	—	**3,949**
BLL 10–24 *μ*g/dL	179	—	226	—	306	—	270	—	334	—	419	—	433	—	557	—	738	—	869	—	764	—	**5,095**
Total with unknown source of exposure																							
BLL ≥25 *μ*g/dL	888	(11.0)	1,588	(17.4)	1,354	(16.6)	714	(10.8)	1,262	(14.8)	1,710	(19.7)	2,151	(23.5)	2,173	(27.6)	1,329	(15.5)	904	(11.0)	742	(10.7)	**14,815**
BLL 10–24 *μ*g/dL	4,096	(35.1)	3,669	(35.7)	3,645	(32.9)	3,190	(28.7)	3,187	(28.1)	2,976	(26.4)	3,877	(29.1)	3,767	(27.5)	7,203	(35.7)	4,565	(23.4)	4,689	(25.2)	**44,864**
**Total adults reported to ABLES (including non–firearm-related exposures)**																							
BLL ≥25 *μ*g/dL	8,049	—	9,142	—	8,154	—	6,584	—	8,525	—	8,691	—	9,151	—	7,863	—	8,561	—	8,196	—	6,927	—	**89,843**
BLL 10–24 *μ*g/dL	11,665	—	10,291	—	11,084	—	11,116	—	11,342	—	11,283	—	13,336	—	13,679	—	20,152	—	19,527	—	18,593	—	**152,068**
**No. of states reporting exposure source** †																							
BLL ≥25 *μ*g/dL	28	—	31	—	33	—	32	—	35	—	35	—	38	—	39	—	39	—	39	—	39	—	**—**
BLL 10–24 *μ*g/dL	10	—	11	—	14	—	13	—	14	—	16	—	19	—	23	—	31	—	30	—	33	—	**—**

**Abbreviations:** BLL = blood lead level; NAICS = North American Industry Classification System.

* Percentage of the total reported per year by BLL group in the relevant category (e.g., in the industry subsector, it represents the proportion exposed at work).

† Fewer states provide work-relatedness and industry data for BLLs of 10–24 *μ*g/dL, compared with BLLs ≥25 *μ*g/dL.

**TABLE 2:** Deficiencies contributing to elevated blood lead levels identified during the investigation of an indoor firing range — CDC’s National Institute for Occupational Safety and Health, California, 2013

Deficiency type	Problem observed
**Engineering control deficiencies**	
Range ventilation system	Airflow at the firing line contained regions of backflow, causing lead to be carried back into the shooter’s breathing zone instead of downrange. The range air supply diffusers produced turbulent jets of air, creating uneven air distribution at the firing line. The downrange airflow was not evenly distributed and did not have the minimum recommended airflow of 30 ft/min (15 cm/sec). The range filters did not have a minimum efficiency reporting value of 18 or 19, so contaminated air was released outside. The range filters did not have side and face gaskets to prevent air from bypassing the filter; this allowed lead-contaminated air to be distributed to other areas served by the ventilation system.
Building ventilation system	Openings in the wall between the firing range and the rest of the building allowed lead to be circulated throughout the building.
**Housekeeping deficiencies**	
Range housekeeping	Carpet and porous materials were present inside the shooting range. Uniforms worn by employees who cleaned the range were reused, laundered infrequently, and stored in an open storage room.
Building housekeeping	Lead was detected on carpets, desks, tables, counters, eating surfaces, and ventilation supply and return air ducts outside the range. It was also detected inside the clean clothing bins and on towels that had been laundered by a commercial launderer. Lead was detected on employees’ shoes as they prepared to leave work. No showering facilities were available for employees. Employees’ hands and street clothes were contaminated with lead.
**Medical surveillance deficiencies**	
Employees	No employees had undergone the required medical surveillance. The physician who evaluated employees to determine their fitness to wear a respirator did not complete the required forms properly.

**TABLE 3:** National Toxicology Program (NTP) conclusions regarding evidence of the principal health effects of low-level lead exposures in adults — United States, 2013

Health area	BLL	Principal health effects	NTP conclusion regarding evidence
Neurologic	<10 *μ*g/dL	Increased incidence of essential tremor	Sufficient
	<10 *μ*g/dL	Psychiatric effects, decreased hearing, decreased cognitive function, increased incidence of amyotrophic lateral sclerosis	Limited
	<5 *μ*g/dL	Increased incidence of essential tremor	Limited
Immune	Unclear	—	Inadequate
Cardiovascular	<10 *μ*g/dL	Increased blood pressure and increased risk of hypertension	Sufficient
	<10 *μ*g/dL	Increased cardiovascular-related mortality and electrocardiographic abnormalities	Limited
Renal	<5 *μ*g/dL	Decreased glomerular filtration rate	Sufficient
Reproductive	<5 *μ*g/dL	Women: reduced fetal growth	Sufficient
	≥15–20 *μ*g/dL	Men: adverse changes in sperm parameters and increased time to pregnancy	Sufficient
	<10 *μ*g/dL	Women: increase in spontaneous abortion and preterm birth	Limited
	≥10 *μ*g/dL	Men: decreased fertility	Limited
	≥31 *μ*g/dL	Men: spontaneous abortion in partner	Limited
	Unclear	Women and men: stillbirth, endocrine effects, birth defects	Inadequate

**Adapted from:** National Toxicology Program. Health effects of low-level lead evaluation. Research Triangle Park, NC: US Department of Health and Human Services, National Toxicology Program; 2013. Available at http://ntp.niehs.nih.gov/go/36443.

## Footnotes

* The OSHA permissible exposure limit for airborne exposure to lead is 50 *μ*g/m^3^ of air for an 8-hour time-weighted average. The standard requires medical monitoring for employees exposed to airborne lead at or above the action level of 30 *μ*g/m^3^, medical removal of employees whose average BLL is ≥50 *μ*g/dL for construction or 60 *μ*g/dL for general industry, and economic protection for medically removed workers, among other things.

† Measures to prevent take-home exposure include showering and changing into clean clothes after shooting or performing firing range maintenance activities, storing clean clothes in a separate bin from contaminated clothing, laundering of nondisposable outer protective clothing by a contractor or by the employer (not by the employee), and leaving at the range shoes worn inside the firing range, or providing disposable shoe coverings.
